# From risk to resilience: a narrative overview of modifiable factors influencing anxiety in children with autism spectrum disorder (part II)

**DOI:** 10.3389/fpsyt.2026.1872455

**Published:** 2026-07-17

**Authors:** Emanuela Elena Mihai, Ileana Ciobanu, Matei Teodorescu, Andreea Georgiana Marin, Koushik Maharatna, Mihai Berteanu

**Affiliations:** 1 Department of Physical and Rehabilitation Medicine, Carol Davila University of Medicine and Pharmacy Bucharest, Bucharest, Romania; 2 Elias University Emergency Hospital, Bucharest, Romania; 3 School of Electronics and Computer Science, University of Southampton, Southampton, United Kingdom

**Keywords:** anxiety, autism spectrum disorder (ASD), children and adolescents, mental health, modifiable factors for anxiety, risk factors

## Abstract

**Background:**

Anxiety disorders are highly prevalent in children and adolescents with Autism Spectrum Disorder (ASD) and contribute substantially to functional impairment and reduced quality of life (QoL).

**Objective:**

This paper represents the second part of a two-part narrative overview addressing internalizing symptoms in ASD. Complementing the first paper focused on depression, the present work examines anxiety, emphasizing shared and distinct modifiable risk and protective factors within a unified conceptual framework.

**Methods:**

A structured narrative overview was conducted, synthesizing evidence from systematic reviews, meta-analyses, and robust clinical studies. Studies were selected based on relevance and scientific rigor, and findings were organized across biological, psychological, and environmental domains.

**Results:**

Anxiety in ASD is associated with multiple interacting factors, including sensory hypersensitivity, intolerance of uncertainty (IU), social challenges, and neurobiological vulnerability. Protective factors such as cognitive-behavioral therapy (CBT), structured physical activity, and supportive environments may mitigate anxiety symptoms and promote adaptive functioning. Nevertheless, variability in study design and population characteristics limits generalizability.

**Conclusions:**

Addressing anxiety in ASD requires a comprehensive, multidisciplinary strategy that integrates risk reduction with resilience-building interventions. Together with the first part of this overview, these findings support transdiagnostic and personalized approach to mental health in children and adolescents.

## 1 Introduction

Autism spectrum disorder (ASD) is a neurodevelopmental condition which presents significant challenges and deficits in social communication and interaction for persons diagnosed with. This condition also encompasses and repetitive and restricted behavioral patterns, and its etiology is multifactorial, thus, showcasing the entwined influence of genetic and environmental factors (1–3). Furthermore, ASD is also linked to major psychiatric comorbidities, anxiety being a prevalent disorder with a significant impact on the clinical aspects of the affected individuals (3). Anxiety and depression are two equally frequent comorbidities which impact young individuals diagnosed with ASD, becoming major public concern issues (3). Mental health disorders (MHD) reflect negatively on the severity of symptoms, functional impairment, quality of life (QoL), and caregiver burden in children and adolescents diagnosed with ASD (2, 4, 5). Therefore, early diagnosis of autism is a crucial step for ensuring timely intervention and improved long-term outcomes, including decreasing symptom severity, functional impairment, quality of life (QoL), and caregiver burden (2).

The multifaceted nature of ASD requires a multidimensional management strategy that incorporates multidisciplinary, biomedical, and behavioral interventions to support overall functioning and well-being. Early diagnosis, adapted therapeutic interventions, as well as the comprehension of aggravating and protective factors, can greatly improve the outcomes both on the short-and the long-term (1, 2, 4, 5).

Despite the growing body of literature examining anxiety in children and adolescents with Autism Spectrum Disorder (ASD), existing research remains largely fragmented and predominantly risk-oriented, with most studies focusing on isolated domains such as prevalence, specific interventions (e.g., cognitive behavioral therapy), or neurobiological mechanisms (2, 3). While these contributions have advanced understanding, they often fail to provide a comprehensive and clinically integrative perspective, particularly with regard to protective and resilience-promoting factors, which remain comparatively underexplored (6, 7). Whereas much of the existing literature has concentrated on identifying vulnerability and risk pathways in ASD, protective influences—particularly those related to rehabilitation, physical activity, psychosocial support, and lifestyle-based strategies—remain comparatively underexplored and often fragmented across studies. These strategies are consistent with current multidisciplinary models of ASD care, which prioritize functional participation, emotional regulation, and long-term well-being, rather than focusing solely on symptom reduction (8–11). Building upon our previous work addressing depression, the present study represents the second part of a two-part narrative overview, extending the same conceptual framework to anxiety. Given the high co-occurrence and shared underlying mechanisms of anxiety and depression in ASD, this complementary approach allows for both focused analysis and coherent integration of internalizing psychopathology. Anxiety and depression frequently co-occur in this population and share overlapping neurobiological, developmental, and environmental determinants (2). However, despite these commonalities, each condition also presents distinct clinical features and intervention needs. For this reason, the two-part structure was adopted to allow focused analysis of each domain, while preserving a coherent and integrative perspective on internalizing psychopathology in ASD.

A narrative overview methodology was purposely chosen, as it is particularly suited to addressing complex, multifactorial conditions such as ASD, where evidence arises from diverse study designs and spans multiple disciplines. Rather than aiming to quantify pooled effects or establish causal inference, this approach enables the integration of heterogeneous findings, the identification of recurring patterns and inconsistencies, and the contextualization of evidence within real-world clinical practice. Studies were selected based on their relevance, methodological soundness, and contribution to advancing a multidisciplinary understanding of modifiable risk and protective factors, interpreted within the broader context of non-modifiable vulnerability markers and individual characteristics, with the ultimate goal of informing more comprehensive, prevention-oriented, and individualized care strategies for anxiety in ASD.

## 2 Materials and methods

This paper represents the second part of a two-part narrative overview, following our previous work focusing on depression in children and adolescents with Autism Spectrum Disorder (ASD). While Part I examined modifiable factors influencing depressive outcomes, the present paper extends this framework to anxiety, acknowledging the frequent co-occurrence and shared underlying mechanisms between these internalizing conditions. Additionally, the modifiable factors are weighted against non-modifiable markers and individual traits of children and adolescents with ASD.

This narrative overview was performed in accordance with established guidelines for narrative reviews. The framework was designed by the multidisciplinary team (E.E.M., I.C., M.T., A.G.M., K.M., M.B.) with entwined experience in education, research, and clinical practice. The narrative overview derived through collaborative discussions based on clinical experience and current evidence, aiming to connect conceptual understanding with empirical data. Different from systematic reviews and/or meta-analyses, which follow predefined established protocols and quantitative synthesis, narrative overviews are guided by a more flexible and interpretative strategy which encompass both the inclusion and contextualization of heterogeneous data presented in systematic reviews, meta-analyses, and robust studies.

### 2.1 Narrative review approach strategy

A narrative review approach was selected to enable the integration of a heterogeneous body of evidence spanning biological, psychological, environmental, and rehabilitative domains. This methodology allows for a broader interpretative and clinically oriented synthesis beyond the constraints of systematic reviews, facilitating the contextualization of findings within real-world healthcare settings. The aim is to support a multidisciplinary, prevention-oriented perspective, emphasizing modifiable risk and protective factors that may improve mental health outcomes and quality of life (QoL) in Autism Spectrum Disorder (ASD) populations.

### 2.2 Search strategy and study selection

A non-systematic literature search was conducted across PubMed, PubMed Central (PMC), Scopus, and Google Scholar up to October 2025. Grey literature was not included in search strategy and selection. The search strategy was adapted from Part I (depression focused overview) and refined to capture anxiety-related outcomes in ASD, ensuring conceptual continuity across the two papers.

Keywords and MeSH terms included: autism spectrum disorder (ASD); anxiety; anxiety disorders; internalizing symptoms; risk factors; protective factors; modifiable factors; mental health; mental health disorders (MHD); resilience; prevention; psychosocial interventions; rehabilitation; rehabilitative strategies; sensory regulation; lifestyle interventions; anxiety in children and adolescents with ASD; children and adolescents. Search terms were adapted to each database to optimize retrieval.

Studies were screened independently by four reviewers (E.E.M., I.C., M.T., A.G.M.), based on title, abstract, and full-text assessment. Discrepancies regarding eligibility and relevance were resolved through discussion with additional reviewers (K.M., M.B.) until consensus was reached. Reference lists of included articles were also screened to identify other relevant studies not captured in the initial search. Consistent with the narrative design, study selection prioritized relevance, methodological robustness, and contribution to conceptual understanding, rather than focusing on exhaustive inclusion of all available studies. A summary of the literature identification and selection process is provided in **Supplementary Figure 1**, while representative search strategies and search dates are presented in **Supplementary Table 1**.

### 2.3 Eligibility: inclusion and exclusion criteria

The multidisciplinary team evaluated the relevance of each paper and also extracted additional references in accordance with the proposed conceptual framework of this narrative overview.

#### 2.3.1 Inclusion Criteria

Studies were eligible if they met the following:

1. Population: children and adolescents (0–18 years) diagnosed with ASD, with outcomes related to anxiety, anxiety symptoms, or internalizing features. Mixed-age studies were included if pediatric data were clearly identifiable.
2. Study design: randomized controlled trials (RCTs), non-randomized trials (NRTs), observational studies (cohort, case–control, cross-sectional), case series, systematic reviews and meta-analyses.
3. Outcomes: clinically diagnosed anxiety disorders, validated scales, or proxy measures of emotional and behavioral functioning.
4. Interventions/exposures: studies evaluating modifiable risk and protective factors, including psychosocial, rehabilitative, pharmacological, sensory, lifestyle-based, and digital interventions.
5. Publication characteristics: peer-reviewed, English-language, full-text articles with sufficient methodological detail.

#### 2.3.2 Exclusion Criteria

Studies were excluded if they:

1. Population: if they focused exclusively on adults or non-ASD populations without separate ASD data.
2. Outcomes: studies not reporting anxiety-related outcomes or focusing solely on other psychiatric comorbidities (e.g., ADHD, depression) without making reference to anxiety symptoms and their entwined nature, given that anxiety, ADHD, and depression are frequently linked.
3. Study design: editorials, commentaries, opinion pieces, conference abstracts without full-data or without reference to empirical evidence, and single case reports with insufficient generalizability.
4. Methodological limitations: papers lacking methodological transparency or relevance to the conceptual framework of modifiable risk and protective factors, or unclearly stated outcomes.
5. Redundancy: duplicate papers or with overlapping datasets. In this case the most comprehensive or recent version was retained in the narrative overview.

Finally, we synthetized evidence from 110 papers regarding the interplay of the risk and protective factors, as well as interventions and strategies for modulating anxiety in ASD children and adolescents. Additional references were cited to provide background information, methodological context, diagnostic definitions, and supporting evidence relevant to the discussion.

### 2.4 Studies’ quality

Given the narrative design, a formal risk-of-bias assessment was not performed. However, emphasis was placed on higher-level of evidence, including systematic reviews, meta-analyses, and randomized trials with and without controls. Study limitations, heterogeneity, and inconsistencies are critically discussed within the manuscript to support balanced interpretation.

### 2.5 Data Synthesis and Interpretation

Due to substantial heterogeneity in study design, populations, and outcome measures, quantitative synthesis was not appropriate for this narrative overview. Therefore, findings were synthesized narratively and organized into thematic domains: biological, psychosocial, environmental, and rehabilitative/modulating factors.

This structure mirrors Part I (depression) to ensure conceptual coherence across the two-part overview, while allowing identification of shared and disorder-specific mechanisms. Particular emphasis was placed on distinguishing risk versus protective factors, as well as exploring their interaction within a multidisciplinary and resilience-oriented framework.

Any limitations and/or discrepancies found across the papers which were included were further discussed by the multidisciplinary team members in order to maintain transparency and academic rigor. The synthesis highlights patterns, inconsistencies, and gaps in the literature, with attention to clinical applicability. This approach supports the development of a comprehensive, integrative model for understanding and managing anxiety in children and adolescents with ASD, complementing the depression-focused findings presented in the first part of this series. This methodological approach focuses on providing a comprehensive perspective of these factors highlighted in the Results and Discussion, as it aims to encompass a synthesis of these factors and the way they interact with each other.

## 3 Results

### 3.1 Risk factors associated with anxiety in ASD children and adolescents

Anxiety is the most common comorbidity among children and adolescents with ASD, with prevalence estimates ranging from 30% to 80% meeting criteria for a clinical diagnosis (12, 13). Anxiety is also a major component of ASD and the incidence of related symptoms in individuals with ASD has been linked to altered interoceptive processing (14). The study of Palser and colleagues investigated whether impaired interoceptive accuracy and exaggerated interoceptive sensibility (subjective sensitivity to internal sensations on self-report questionnaires) in autistic adults, could be extended into a school-age sample of children and adolescents (n = 75), finding that interoceptive sensibility was the best predictor of anxiety symptoms in the sample. Autistic children and adolescents showed lower metacognitive accuracy for interoception in comparison to their non-autistic peers, but the autism group reported higher confidence than the typical group in the discrimination task (14). These findings were also consistent with theories of ASD as a disorder of interoceptive processing, and additionally showcased the importance of validating cognitive models of developmental disorders within developmental populations.

### 3.2 Implications and interplay of genetic and neurobiological risk factors

Growing body of evidence suggests that autism spectrum disorder (ASD), major depressive disorder, and other psychiatric disorders share overlapping genetic risk factors (3, 15). Individuals with ASD may face a more elevated risk of developing anxiety, clinically diagnosed depression, and bipolar disorder when compared to age- and sex-matched persons (16).

Neurobiological vulnerability plays a crucial role as a contributor to anxiety in ASD. Research data points out the role of altered pathways to neurotransmitter systems (notably for dopamine and serotonin), amygdala overactivation, and prefrontal cortex dysregulation in shaping emotional reactivity (17, 18). For example, findings highlight autonomic arousal pathways, sensory sensitivities, and familial aggregation as meaningful factors contributing to anxiety (17, 18). Moreover, high evening levels of cortisol and flattened diurnal rhythms in adolescence have also been associated with increased anxiety risk (19).

### 3.3 Intelligence quotient (IQ) and verbal IQ (VIQ) and concurrent physiological factors: age, menarche, puberty

Anxiety symptoms were highly associated with a higher IQ. High-functioning individuals with ASD are at increased risk for anxiety due to greater awareness of social limitations and increased expectations (20, 21).

As shown in various studies, there is a broad range of individual characteristics which can modulate anxiety levels in ASD. Regarding age and puberty, adolescents experience more anxiety due to biological and social changes. The “two-hit” model describes puberty as a second blow to already vulnerable neurodevelopmental systems (19, 22). Evidence indicate that anxiety symptoms in children with ASD may intensify with age (23, 24).

Compared to males, females tend to internalize distress, and therefore, anxiety symptoms are often missed due to better social camouflage techniques (25). Female gender and higher ASD severity predicted more anxiety and depression symptoms (26). Also, male-typical externalizing behaviors tend to be more disruptive both at home and school when compared to female-typical internalizing behaviors, therefore prompting assessment and diagnosis for boys, notably in comparison to high-functioning girls (26). High rates of anxiety have been reported in girls with ASD and/or ADHD (25).

Young individuals with concurrent ASD/gender dysphoria (GD) were at significantly greater risk of suffering from anxiety when comparing them to youth with ASD alone, GD alone, or neither of the two diagnoses (27).

### 3.4 Emotion regulation, social interaction, bullying, alexithymia, and intolerance of uncertainty

Data showed that adolescents with ADHD who had higher scores on the behavioral inhibition system (BIS), suffered from comorbid ASD, and faced bullying reported a much higher severity of anxiety and depressive symptoms (28). BIS traits, ASD, and bullying were highly correlated with anxiety and depression (28). Besides other factors, victimization alone was correlated with greater levels of anxiety, depression, and also suicidality (29).

Concerning loneliness and social interaction, evidence indicated that children and adolescents with ASD face greater levels of loneliness compared to neurotypical peers. Moreover, loneliness is moderately to strongly linked to both anxiety and depression (30).

A personality trait, alexithymia is characterized by difficulties in identifying and expressing emotions and it is commonly observed in adults with ASD, being linked to deficits in emotion recognition and empathy. One study explored its presence in younger individuals, finding significantly higher rates in adolescents with ASD (55%) compared to their non-ASD peers (16%) (31). Among those with ASD, alexithymia was associated with greater self-reported anxiety, parent-reported emotional difficulties, sensory processing differences, and poorer emotion recognition, but not with theory of mind abilities. These findings indicate that alexithymia is highly prevalent in adolescents with ASD and is linked to specific emotional and cognitive challenges (31).

Emotional dysregulation and limited emotional insight are strong predictors for anxiety, mainly in young population with coexisting sensory (32). Alexithymia was associated with increased self-reported anxiety, it is highly prevalent, and has selective cognitive correlates in young individuals with ASD (31). Social anxiety in autistic and neurotypical adolescents shares similar underlying mechanisms, including intolerance of uncertainty (IU), alexithymia, maladaptive emotion regulation, and sensory hypersensitivity. These factors, particularly IU, alexithymia, and sensory hypersensitivity, mediate the link between autistic traits and social anxiety, highlighting potential transdiagnostic targets for intervention approaches (33).

Social support plays an important role in shaping anxiety level in ASD children. Although positive parenting approach and peer relationships can mitigate anxiety, poor parental mental health and lack of social reciprocity can exacerbate it (22).

In the study by Leachman et al., participants within the high-anxiety cluster reported the most severe depressive symptoms on the Self-Description Questionnaire I (SDQ-I), indicating that depression may serve as a key marker of anxiety in children and adolescents with ASD. Among the anxiety-related measures, peer problems emerged as the strongest predictor of cluster membership (13). Anxiety in children with ASD is closely linked to family stress, mood disorders, and depression. Notably, nearly all participants with an ASD diagnosis (94%) were classified within the moderate to high anxiety clusters (13).

Intolerance of uncertainty (IU) is a trait-like risk factor characterized by maladaptive responses to uncertain situations (33, 34). Emerging evidence suggests that IU is heightened in youth with ASD and shows a positive association with anxiety, identified as a dispositional risk factor for anxiety (17, 24). Autistic traits and intolerance of uncertainty (IU) were directly linked to anxiety symptoms (17). Evidence also indicate a potential link between intolerance of uncertainty and separation anxiety (24). Data also showed that IU is directly linked to ASD features possibly explained through common genetic, neurological, or psychological foundation (34).

### 3.4 Sleep disorders, sensory sensitivity and mood disorders

Multiple evidence from studies show that sleep fragmentation, delayed sleep phase, and sensory hyper- or hypo-responsivity are associated with increased anxiety (35, 36). Other features such as sleep onset latency, reduced total sleep, and poor subjective sleep quality are closely associated with both anxiety and functional impairment (20, 37). Depression often co-occurs with anxiety, notably during adolescence, and symptoms of both can be worsened by poor sleep and sensory dysregulation (3, 38).

Given that sleep is both a behavioral and emotional regulator, sleep fragmentation or deprivation can increase behavioral disturbances in children with ASD, therefore, possibly being a trigger factor for disruptive/inflexible behavior and anxiety (39). Data from one study showed that insomnia is a significant risk factor for the development of anxiety disorders (40). Additionally, anxiety was linked to all types of sleep problems in ASD children (i.e.; bedtime resistance, delay of sleep-onset, duration of sleep, sleep-related anxiety, and night wakings as shown by Mazurek (41). Data showed that in the youth with ASD sleep anxiety, co-sleeping, night waking, and parasomnias showed a decrease with age, as seen in longitudinal trends in non-ASD youth (42). One study from Sakamoto found that “anxious/depressed” scores on the Child Behavior Checklist (CBCL) were linked with low registration and sensory avoiding on the Sensory Profile 2 (SP2) (43). Sleep-related problems (SRPs) have been shown to improve following cognitive-behavioral therapy (CBT) in both youth with ASD and those without, and a plausible explanation lies in the links between ASD symptoms, anxiety, and SRPs. Modularized CBT, adapted to address the specific needs of youth with ASD and anxiety, has demonstrated efficacy in reducing anxiety symptoms and treatment gains have been linked not only to decreases in anxiety levels but also to reductions in ASD-related social and behavioral symptom severity and associated functional impairments (44–47).

Data indicated that sensory features, specifically sensory avoidance and sensitivity, mediate the relationship between autistic traits and anxiety symptoms in boys with ASD. Higher levels of sensory avoidance and sensitivity were associated with increased anxiety levels (48).

### 3.5 Co-occurring health issues and mental health conditions

Data showed increased rates for anxiety and depression, and social phobias in deaf and hard-of-hearing children and adolescents with co-occurring ASD. The prevalence of affective disorders in auditory-impaired children and adolescents is similar to estimates of prevalence for those individuals with normal-hearing (49).

Children and adolescents with ASD are at high risk for specific anxiety disorders, namely elevated levels of impairments regarding social motivation and communication, important indicators for comorbid disorders, in particular social anxiety and ASD (50).

Psychiatric comorbidities are highly prevalent among children with autism spectrum disorder (ASD), with approximately 70% presenting with at least one additional diagnosis and 41% meeting criteria for two or more (51). The most commonly identified conditions were social anxiety disorder (29.2%), attention-deficit/hyperactivity disorder (ADHD; 28.2%), and oppositional defiant disorder (28.1%) (51).

Anxiety rarely occurs alone in ASD and is often intensified by attention-deficit/hyperactivity disorder (ADHD): Autistic individuals with comorbid ADHD report more severe generalized and social anxiety (18, 52). Depression often co-occurs with anxiety, notably during adolescence, and data showed that the symptomatology of both mood disorders can be exacerbated by poor sleep quality and sensory issues and dysregulation (3, 38).

Evidence suggests that the population at high familial risk for suffering from ASD is also susceptible to develop anxiety, ADHD, and other developmental conditions. Nevertheless, ASD and anxiety are difficult to distinguish from in early childhood, and special attention is highly needed for better outcomes both on the short- and long-term (53).

Given that ASD and deafness may both confound or delay each other’s diagnosis, data from scientific literature highlights the increased comorbidity noticed between these two conditions, yet the significance of this correlation is still unclear (49). Even though data points out that auditory impairment is not likely to be an etiological factor in autism, hearing impairment may be considered a marker for brain damage in ASD (49).

Other preexisting conditions including asthma, epilepsy, and gastrointestinal issues may exacerbate physiological stress responses (54, 55). Visually impaired adolescents have higher levels of anxiety in comparison with normal-sighted adolescents (56). One study yielded preliminary evidence in the favor of a connection between migraines, headaches, sensory hyperreactivity, and anxiety symptomatology in ASD, suggesting strategies for subtyping and exploring a common pathogenesis of these features (57).

Evidence indicated that 84% of the youths with ASD display levels of anxiety high enough to interfere and impair their daily function (6). Data showed that chronic exposure to stress in childhood and adolescence can result in lasting structural changes in the developing brain, potentially disrupting social behavior and heightening the risk of future psychopathology (58, 59).

### 3.6 External factors: restrictions during pandemic, life stress and trauma

A systematic review including data from over 55, 000 children and adolescents all over the world (with mean age 11.3 years old) indicated that the pandemic had a significant impact on high rates of emotional and behavioral difficulties for anxiety, along with depression, irritability, and anger (60).

Environmental factors shape anxiety outcomes in ASD in a significant manner, including socioeconomic status (SES) and low SES is correlated with limited access to services and chronic exposure to stress (3). Additionally, life stress and trauma, including bullying, academic pressure, and emotional neglect are frequently identified as contributing factors for anxiety (61).

The findings suggest that adolescents with ADHD who exhibit higher behavioral inhibition system (BIS) scores, present with comorbid ASD, and experience bullying victimization are more likely to report heightened anxiety and depressive symptoms (28). Among young individuals with ASD, older age and more severe oppositional defiant disorder (ODD) symptoms were significantly linked to both cyberbullying victimization and perpetration. Notably, victimization alone was uniquely related to elevated levels of depression, anxiety, and suicidality (29). For perpetrator-victim, adolescents with high functioning ASD who both bullied and were bullied (perpetrator-victims) experienced the most severe depression and anxiety (62).

### 3.7 Other relevant findings

-Eating Behavior(s) and Gut Imbalance: Sensory profiles related to food texture and preferences are associated with anxiety and daily functioning (63). A connection between gut-brain interactions and mood regulation is suggested, though further research is needed and more longitudinal designs are required.

-Symptom Severity and Diagnostic Challenges: Anxiety occurs across the ASD spectrum. High-functioning individuals may experience social comparison distress, while those with greater impairment struggle with unpredictability and transitions (64).

-Camouflaging Behavior: Particularly among females and cognitively able youth, masking leads to internalized stress and elevated long-term anxiety (64).

-Measurement Limitations: Difficulty expressing anxiety verbally can lead to underdiagnosis. Some children exhibit somatic symptoms or behavioral outbursts in response to anxiety rather than expressing verbal concern (3).

-Early ASD characteristics: A non-controlled study highlighted the early ASD characteristics linked to the severity of anxiety symptoms in adolescence. Specifically, elevated anxiety in adolescence were associated with the levels of cognitive ability, adaptive functioning, and restricted and repetitive behaviors (RRBs) observed in toddlerhood (65).

-Stereotypical behaviors: These behaviors are identified as major predictors for anxiety in the ASD population aged 3 to 6 years old included in the study of Duran-Bouza et al.

(24).

-Sensory hyper- and hyporeactivity: Evidence indicated that sensory hyperreactivity and hyporeactivity might play a role in specific anxiety symptoms (66).

**Figure 1:**
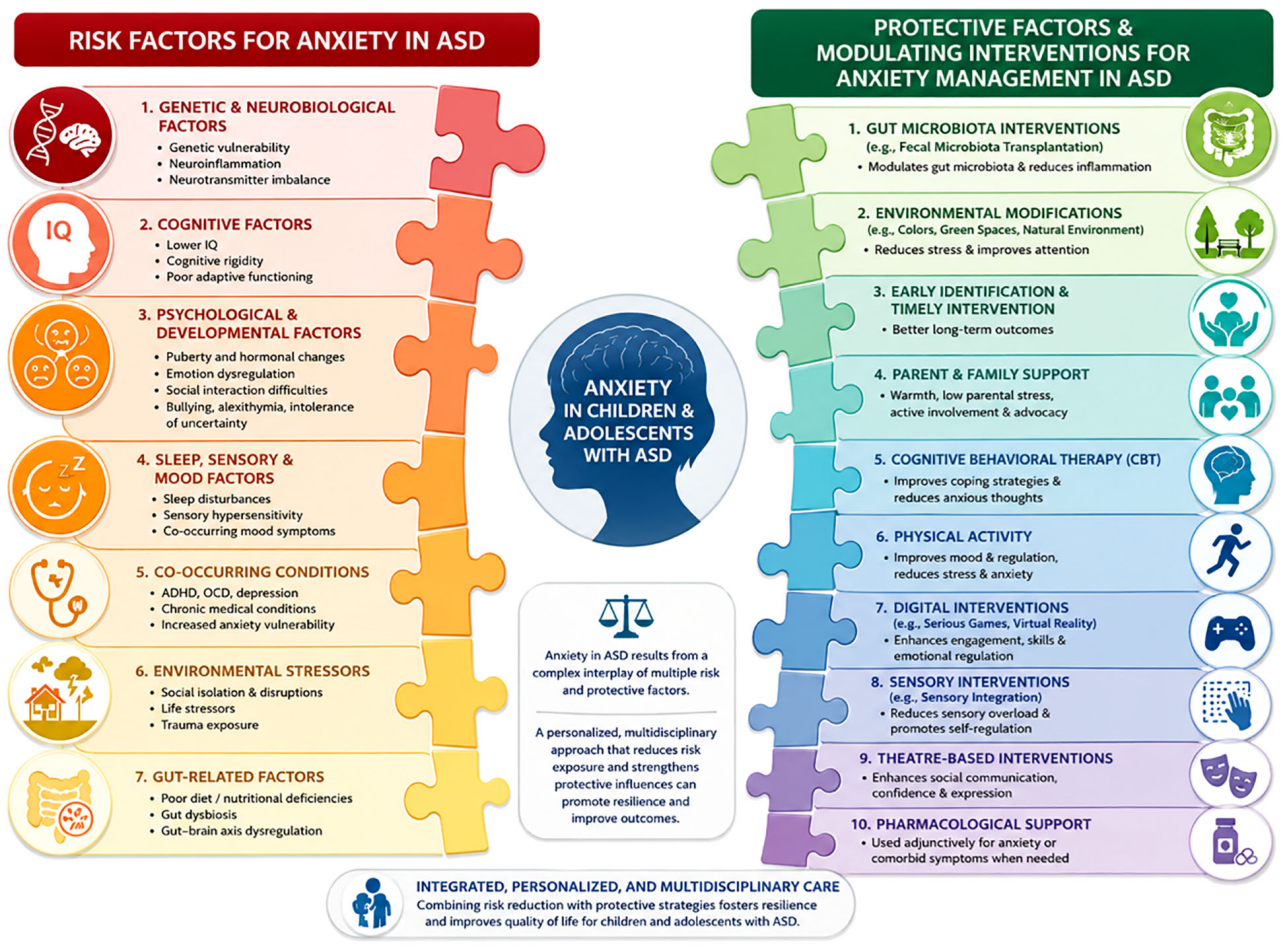
Conceptual overview of risk and protective factors influencing anxiety in children and adolescents with Autism Spectrum Disorder (ASD). The image summarizes key factors identified in the reviewed literature that may contribute to improved mental health outcomes in this population. It was conceptually designed by the authors based on the evidence synthesized in this review (references 1, 3, 6, 9, 15–120). An AI-assisted tool was used for the initial visual generation, followed by manual review and refinement by the authors, who take full responsibility for the content and accuracy of the figure. ASD, Autism Spectrum Disorder; CBT, Cognitive Behavioral Therapy; PA, Physical Activity; SNRIs, Serotonin–Norepinephrine Reuptake Inhibitors; SSRIs, Selective Serotonin Reuptake Inhibitors.

**Figure 1** highlights a conceptual overview on the role and interplay of various factors (risk and protective factors, as well as modulating interventions) interfering with anxiety in children and adolescents diagnosed with ASD. This schematic illustrates a conceptual, integrative model of anxiety in children and adolescents with Autism Spectrum Disorder (ASD), highlighting the dynamic balance between risk factors and protective/modulating interventions. The left side presents a range of interacting vulnerabilities—including genetic and neurobiological predispositions, cognitive and developmental characteristics, emotional and social challenges, sensory sensitivities, comorbid conditions, and environmental stressors—that may contribute to the onset and persistence of anxiety. In contrast, the right side depicts protective factors and targeted interventions, such as early diagnosis, family support, cognitive-behavioral therapy (CBT), physical activity, sensory-based strategies, environmental modulation, and selected pharmacological approaches, which can mitigate anxiety and promote resilience. Non-modifiable vulnerability factors should not be considered intervention targets but they remain clinically important as potential contributors to risk stratification and may inform individualized prevention, monitoring, and support strategies.

The puzzle-like structure symbolizes the multifactorial and interconnected nature of ASD, where individual elements interact within a broader system. At the center, anxiety is conceptualized as the result of this continuous interplay, emphasizing the importance of a personalized, multidisciplinary approach that reduces risk exposure while strengthening protective mechanisms to improve mental health outcomes.

## 4 Protective factors for anxiety in ASD

Despite the overwhelming period of isolation during COVID-19 pandemic and the negative effects on anxiety, depression, irritability, and anger, evidence showed that the presence of any kind of family routines and good parent-child communication were identified as protective factors (60).

Growing evidence indicated that anxiety symptoms in children with ASD are likely to intensify with age, therefore, is of paramount importance to focus on early interventions to tackle anxiety and its symptoms and prevent its exacerbation on the long-term (23, 24, 67). Children with ASD may be particularly vulnerable to separation anxiety, given the central role of parental support in facilitating their understanding of and adaptation to daily challenges. Most often, children with autism rely on their parents to manage uncertain circumstances, and their parent support and adaptation to new challenges may improve their response (24, 68). Extending early interventions to younger children has the potential to substantially improve their quality of life (QoL) and of their families. Early adoption of strategies targeting uncertainty may mitigate anxiety and promote the development of emotional resilience in children with ASD (24, 68).

### 4.1 Fecal microbiota transplantation

Given the high prevalence of gastrointestinal disturbances and gut microbiota alterations in children and adolescents with ASD, fecal microbiota transplantation (FMT) has emerged as a promising area of investigation. Current evidence suggests that FMT may improve gastrointestinal symptoms, sleep disturbances, and certain behavioral manifestations associated with ASD. Delivery through upper gastrointestinal routes, such as oral capsules or nasojejunal (NJ) tube administration, has been reported to demonstrate favorable efficacy and safety profiles compared with lower gastrointestinal delivery methods (69).

In an open-label study involving 38 children with ASD, oral lyophilized FMT was associated with improvements in gastrointestinal symptoms, sleep disturbances, and selected ASD-related behavioral outcomes, accompanied by changes in gut bacterial and fungal microbiota composition (70). Similar findings have been reported in other studies, with proposed mechanisms involving the modulation of gut microbial communities and restoration of gut–brain axis functioning (71).

Although preliminary findings suggest potential benefits for emotional and behavioral regulation, including reductions in anxiety-related symptoms, the current evidence remains limited and largely indirect. Most studies have focused primarily on gastrointestinal symptoms, sleep, behavior, and overall ASD-related outcomes rather than anxiety as a primary endpoint. Therefore, FMT should currently be considered an emerging and experimental area of research with potential relevance for anxiety prevention and management in ASD, rather than an established intervention specifically targeting anxiety symptoms (72).

### 4.2 Colors, green spaces, natural environment

Green space demonstrated a protective influence on mental health. Among 41 studies reviewed, the majority (70%, n = 29) reported that neighborhood green spaces had beneficial effects on the mental health of vulnerable populations, while 17% (n = 7) found no significant association, and 12% (n = 5) reported negative effects (73). Bray et al. reported that walking in or simply being exposed to green spaces leads to immediate improvements in mood and reductions in state anxiety. Evidence from non-randomized and observational studies suggests that social interaction, physical activity, and mindfulness mediate the link between green space exposure and mental health benefits. The authors propose that the restorative qualities and relative quietness of green environments foster mindfulness and disrupt rumination, thereby reducing the risk of anxiety and depression. Their review and conceptual framework provide valuable guidance for healthcare professionals, highlighting the therapeutic potential of nature contact and the role of green social prescribing (74). The visual environment is particularly important as it can worsen or lighten ASD symptoms, including anxiety. A study provided valuable results by applying a survey which highlighted valuable details into the preliminary considerations for better designing and adapting an engaging, relaxing, and welcoming indoor environment that can provide the users with positive and regulatory sensory experiences and decreases anxiety levels (75). Dark and intense colors are the least preferred. The relation of light with color is of paramount importance, as they are linked. In combination, both factors play a major role in creating the right environment for children and adolescents with ASD suffering from anxiety (75).

Beyond diet, environmental factors such as exposure to nature may also support mental health in children and adolescents. Lomax emphasized the beneficial effects of natural environments on well-being, although evidence was mixed for certain outcomes, including self-concept, problem-solving, mood, emotional well-being, self-esteem, and depression (76). Among two cohort studies reviewed, one confirmed nature’s positive influence on improving mental well-being, reinforcing the need to integrate environmental and lifestyle interventions alongside dietary strategies for holistic support in pediatric populations (76).

### 4.3 Early ASD diagnosis and intervention for the management of anxiety

Early ASD diagnosis (ages 2–5) allows children to access timely interventions that support communication, social interaction, and motor development during a period of heightened neuroplasticity (1). Evidence shows that early intervention significantly improves cognitive, language, and social-emotional outcomes, enhances long-term social functioning and independence, and reduces the need for intensive services, lowering overall healthcare costs (1, 77, 78). Early diagnosis also enables entry into specialized educational programs that improve academic progress and socialization (1, 79). Collectively, studies highlight early diagnosis as crucial for better developmental outcomes and reduced family stress (1, 77–79). Elevated levels of difficulty in sensory processing and restricted and repetitive behaviors (RRB) in early childhood are found to be potential risk factors for later anxiety disorder in children diagnosed with ASD (80). The findings highlight the need to individualize prevention and treatment to optimize anxiety disorder outcomes in children with ASD. Additionally, more studies are needed to assess the efficacy of implementing early, specific, and tailored treatment for anxiety in children with ASD (80).

### 4.4 Parent characteristics

Pregnancy complications were specifically associated with impairment related to social anxiety and schizoid personality symptoms. Intellectual functioning (IQ) uniquely predicted impairment linked to schizophrenia, ASD, and oppositional defiant disorder symptoms (81). Lower maternal education was associated with increased odds of impairment in social anxiety, depression, aggression, and mania, as well as with a greater overall number of impairing conditions. Season of birth emerged as the most consistent correlate of symptom-induced impairment according to teacher ratings, though not parent ratings. Children born in the fall demonstrated higher rates of co-occurring psychiatric and ASD-related impairments, along with a greater total number of impairing conditions. These findings suggest that several risk factors previously associated with symptom severity are also strongly linked to functional impairment (81). One study’s results indicated that mothers of children with ASD and anxiety tended to face higher levels of anxiety compared with mothers of children with ASD-only. These findings could be explained by the expression of a genetic and/or familial vulnerability to develop anxiety (80).

### 4.5 Serious games

Serious games have shown strong potential for enhancing social and emotional functioning in autistic individuals. A systematic review by Carneiro et al. reported that serious-game–based interventions improved key social-communication domains, including emotion recognition and decoding, emotional regulation, eye gaze, joint attention, and broader behavioral skills (82). Complementing these findings, another systematic review demonstrated that game-based tools—designed not only for entertainment but also for targeted therapeutic or educational purposes—yielded significant benefits across diverse social-skills outcomes (83). Of the 104 criteria evaluated across the included studies, 57 showed measurable improvement, with 22 studies reporting significant gains in at least one domain and 13 studies demonstrating improvements across all measured outcomes (83). The study of Ma and the colleagues showed that the intervention treatment of experimental interactive virtual reality-motion (VR-Motion) serious games through pre-experiment and formal experiment has scored a positive effect on decreasing both anxiety and depression in children with ASD (84). Evidence showed that experimental groups scored better results compared to the control group, showcasing the game’s efficacy in improving levels of social interaction, anxiety and depression levels (84). Collectively, the evidence strongly supports serious games as an effective, engaging, and scalable method for teaching social skills and supporting psychosocial development in autistic individuals (83, 84).

**Table 1** highlights and summarizes protective factors for anxiety in ASD children and adolescents based on level of evidence: systematic reviews and/or meta-analyses, overviews, narrative reviews, randomized controlled trials, clinical trials, case studies, national surveys. The table presents protective factors, mechanism description, and the domain targeted based on level of evidence of the papers included in this narrative overview.

**Table 1:** Summary of protective factors for anxiety in ASD children based on level of evidence.

Protective factor	Description mechanism	Target domain	Level of evidence
Fecal Microbiota Transplantation (FMT)	Improve core symptoms of ASD, gastrointestinal disturbances, and sleep problems in affected children.	Gastrointestinal and behavioral regulation; decrease anxiety levels	Systematic reviews/Meta-analyses/Open-label study (69–72)
Digital Health Interventions (virtual reality (VR), serious games)	Improve accessibility, enhance engagement, monitoring, and ensure continuity of care	Improve social skills, decrease anxiety level and foster emotional regulation	Systematic reviews/Randomized controlled trial (82-84)
Cognitive Behavioral Therapy (CBT)	Targets anxiety, emotional regulation, and maladaptive thought patterns	Modulate anxiety, depression and develop coping skills	Systematic reviews/Meta-analyses/Randomized controlled trials/Non-randomized controlled trials, (35, 44, 88-105)
Sensory Integration Therapy (e.g.: sensory integration, environmental modulation) and Theatre-based Therapy	Addresses sensory processing difficulties to improve participation and behavior	Sensory modulation and better daily functioning	Systematic reviews/Meta-analyses/Randomized controlled trials (107-113)
Structured Exercises & Physical Activity (including martial arts, yoga, horseback riding etc.)	Enhances motor skills, emotional regulation, self-esteem, and social interaction	Improve motor skills, mental health, increase and facilitate socialization	Systematic reviews/Meta-analyses/Randomized controlled trials/National survey (9, 91-106)
Use of Colors (Natural environment, green spaces)	Can influence sensory comfort, boost attention, and ease emotional regulation	Facilitate sensory regulation, behavior	Systematic review/Systematic review and conceptual framework/Meta-review/Case study (73–76)
Pharmacological Treatment (e.g.: SNRIs: Serotonin–Norepinephrine Reuptake Inhibitors; SSRIs: Selective Serotonin Reuptake Inhibitors)	Reduces severe irritability, aggression, anxiety, or depressive symptoms when clinically necessary	Facilitate behavioral regulation and mood changes, decrease anxiety and depression	Systematic review and network meta-analysis/Overview/Narrative reviews (114-120)

Overall, the strength of evidence supporting protective factors and interventions for anxiety in children and adolescents with ASD varies considerably across domains (**Table 2**). The most robust evidence currently supports cognitive behavioral therapy (CBT), which has been evaluated in multiple systematic reviews, meta-analyses, randomized controlled trials, and non-randomized controlled studies. Structured physical activity and exercise interventions also demonstrate consistent benefits across systematic reviews and controlled studies, particularly regarding emotional regulation, social functioning, and anxiety reduction.

**Table 2:** Interventions and pharmacological treatment based on the strength of evidence .

Intervention	Evidence base	Relative strength of evidence
Cognitive Behavioral Therapy (CBT)	Systematic reviews, Meta-analyses, Randomized controlled trials, Non-randomized controlled trials	Strong
Structured Physical Activity (PA)	Systematic reviews, Meta-analyses, Randomized controlled trials,	Moderate to Strong
Digital interventions	Systematic reviews, Randomized controlled trials,	Moderate
Sensory integration/theatre-based	Systematic reviews, Meta-analyses, Randomized controlled trials	Moderate
Environmental modifications/colors	Reviews, Conceptual frameworks, Case studies	Preliminary
Fecal microbiota transplantation (FMT)	Systematic reviews, Meta-analyses, Open-label studies	Emerging/Preliminary
Pharmacological treatment	Reviews, Network meta-analysis	Moderate for selected symptoms; limited for anxiety-specific outcomes

Emerging evidence further supports the potential role of digital health interventions, sensory-based approaches, and environmental modifications; however, findings remain more heterogeneous. In contrast, interventions such as fecal microbiota transplantation (FMT) and certain complementary therapies remain preliminary and require additional high-quality studies before firm conclusions can be drawn regarding their effectiveness for anxiety-specific outcomes in ASD populations.

## 5 Implications for parents and caregivers

A significant association emerged between parenting stress and the child’s ASD diagnosis, with parents of children with autism reporting substantially higher stress levels (85). Follow-up analyses also identified a small positive correlation between parenting stress and maternal age, indicating that older mothers experienced greater stress. No other significant associations were observed. Given these findings, maternal age and the child’s ASD diagnosis were retained as covariates in subsequent analyses. Parenting stress was positively correlated with parental anxiety and child behavior problems, while being negatively associated with parenting self-efficacy. These results suggest that ASD symptomatology significantly shapes the parenting experience, particularly by increasing stress and influencing maternal perceptions of parental competence (85).

Supplementation of folic acid at suitable levels around the time of conception seems to be a protective factor towards autism and may ameliorate the impact of toxic chemicals (86).

A systematic review and meta-analysis reported that parents whose children received an early ASD diagnosis experienced lower levels of stress and anxiety (1, 87). Early identification enabled families to access appropriate services and support sooner, which in turn helped reduce parental stress and contributed to improved overall family functioning (1).

## 6 Interventions for modulating anxiety in children with ASD

### 6.1 Cognitive behavioral therapy

Evidence placed CBT as a highly effective intervention for the management of anxiety, depression, and other related symptoms in children and adolescents, as well as for those diagnosed with ASD.

A meta-analysis of 19 RCTs (n=833) from Sharma et al. showed large effects on clinician-rated anxiety and moderate effects on parent- and self-reported measures, though gains were not maintained at follow-up. Additionally, younger children and individual therapy formats showed greater benefits (88). In 87 studies (n=5964), CBT increased remission of primary anxiety diagnoses (49.4% vs. 17.8% controls) and all anxiety disorders. Effects were consistent across individual and group formats and samples with or without ASD (89). Across 93 systematic reviews (393 RCTs), behavioral interventions incorporating CBT improved sleep parameters and related mood disturbances in children with insomnia, ADHD, and ASD, though overall evidence quality was low (35). A CBT intervention in 40 participants produced significant clinical improvements post-treatment as shown by Nadeau (44). In a randomized clinical trial including 167 children diagnosed with ASD and maladaptive and interfering anxiety, there were two variants of CBT compared with a treatment-as-usual group. Therefore, CBT designed for children diagnosed with ASD showed significantly lower anxiety scores on the primary endpoint compared to standard-of-practice CBT and treatment-as-usual. however, both types of CBT scored better rates of positive response to treatment compared to usual treatment scheme (90).

Nevertheless, based on evidence from multiple meta-analyses and clinical trials, CBT is effective to consistently improve anxiety levels, depression and depressive symptoms, sleep difficulties, and stress in children and adolescents, as well as for those diagnosed with ASD. Tailored and well-planned interventions offer the best benefits, placing CBT as a cornerstone in mental health rehabilitation of children and adolescents (35, 44, 88–90).

### 6.2 Physical activity and structured interventions for anxiety in ASD children and adolescents

Structured exercise and broader forms of physical activity (PA) have been associated with meaningful improvements in behavioral, cognitive, social, and emotional functioning among individuals with ASD. The study of Toscano, as well as a large systematic review and meta-analysis of 59 studies of Liu et al. found that PA interventions yielded notable improvements in overall mental health, cognitive function, psychological well-being, and reductions in internalizing and externalizing problems (91, 92).

Physical activity also plays a crucial role in motor and fundamental movement skills (FMS). Ji et al., in a meta-analysis, showed that exercise significantly improved locomotor skills, object control skills, and stability skills in children with ASD, with locomotor outcomes demonstrating particularly large effect sizes. These improvements support not only physical but also cognitive and social development, laying a foundation for an active lifestyle (93). Pan added evidence that water-based programs such as swimming not only improved aquatic skills but also showed potential in enhancing social abilities (94). One study emphasized the role of structured sports programs, including roller-skating, aquatic activities, and judo, in improving emotion regulation, self-esteem, motor skills, and social behaviors, with technical and game-centered approaches offering cognitive and social benefits (95). Similarly, a systematic review including findings from eight studies indicated that interventions such as yoga, community-based football programs, app-supported walking, group exercise, and horseback riding are associated with reductions in anxiety among individuals with autism. Overall, the evidence synthesized suggests moderate to strong support for the role of physical activity in alleviating anxiety symptoms in both children and adults diagnosed with ASD (96).

Sowa and Meulenbroek reported that while all physical activity programs produced significant gains, individualized interventions led to greater improvements in both motor performance and—unexpectedly—social skills. Despite small sample sizes, their findings suggest that children and adults with ASD derive the most benefit from one-to-one exercise programs, particularly regarding motor and social functioning (97). Similarly, a systematic review and meta-analysis focusing on PA and functioning, found that PA interventions produced substantial improvements in manipulative and locomotor skills, skill-related fitness, social functioning, and muscular strength and endurance (9).

A systematic review reported that aquatic interventions delivered by professionals trained in both behavioral support and aquatic therapy significantly enhanced motor abilities and social engagement while reducing autistic behaviors in children with ASD. Their results highlight the value of structured, multidisciplinary aquatic programs as part of ASD rehabilitation (98).

PA is an important contributor to emotional and psychological well-being in individuals with ASD. Evidence from one study indicates an inverse relationship between levels of physical activity and symptoms of anxiety and depression in youth, including those with ASD; however, elevated rates of comorbid anxiety and depression remain prevalent in this vulnerable population (99).

Evidence from 32 studies showed that physical activity and movement-based interventions can positively influence neural activity and behavior in developmental disabilities, with chronic programs producing greater effects than single sessions (100). Neuroimaging findings (EEG, fMRI, DTI, fNIRS) highlight benefits such as normalized cortical arousal in ADHD and stronger social brain connectivity in ASD. For children with ASD, martial arts–based training (e.g., Nei Yang Gong) improved memory functions, recall, and retrieval strategies, while theatre-based movement programs improved recognition of faces. These interventions suggest that martial arts and similar structured physical practices promote neural plasticity, strengthen executive and memory functions, and support social cognition (100). In general, martial arts offer therapeutic potential beyond physical fitness, with implications for improving cognitive development and social interaction in children with ASD. More standardized, large-scale research is needed to consolidate these findings and guide clinical application.

Through a sociological survey it was also sought to examine whether combat sports could serve as means of rehabilitation and motor development for individuals with ASD, whereas the majority of respondents (77%) indicated that combat sports could be effectively integrated into rehabilitation and motor development programs (101). Additionally, through martial arts, it was noted a gain both in confidence and strength, as well as for movement control and coordination (101). Martial arts–based interventions, encompassing both internal and external styles, demonstrated significant positive effects on ASD-related symptoms, including social interaction and communication skills, self-regulation, memory, postural control, and cognitive functioning. Reported effect sizes ranged from moderate to large (102). After 26 sessions, parents of boys with ASD who participated in the martial arts intervention reported a significant increase in positive social behaviors and a significant reduction in negative social behaviors compared to the control group from pre- to postintervention (103). Tabeshian et al. reported that participation in Tai Chi Chuan led to a approximatively 25% reduction in stereotypic behaviors, as measured by the Gilliam Autism Rating Scale-2 (104). Notably, these behavioral improvements were sustained at follow-up, indicating lasting benefits beyond the intervention period. Sarabzadeh et al. conducted 18 sessions for a period of six weeks of Tai Chi Chuan program (60 minutes per session) and observed significant improvements in ball skills and balance performance, though no significant changes in manual agility were found. The authors attributed these effects to the slow, focused, and disciplined nature of Tai Chi movements, which enhance motor control and body awareness. Therefore, Tai Chi Chuan appears to be an effective therapeutic approach for reducing motor limitations and supporting functional skills in daily life among children with ASD (105).

A systematic review and meta-analysis showcased that exercise interventions were associated with a significant reduction in comorbid anxiety and among the different modalities applied, aerobic exercise showed the strongest anxiolytic effect exceeding that of other forms of activity. Additionally, beneficial effects were observed on core ASD symptoms and attention deficits related to ADHD (106). In contrast, findings regarding sleep outcomes were inconsistent, likely due to substantial heterogeneity across studies. Synthesis concluded that exercise, notably those focused on aerobic interventions, are an effective non-pharmacological therapy to manage anxiety and affective dysregulation in children diagnosed with ASD. These findings highlight the importance of integrating aerobic exercise into clinical treatment plans to improve emotional well-being and QoL in children with ASD (106).

### 6.3 Sensory interventions (e.g. sensory integration and environmental modulation)

A meta-analysis of eight studies (n=426) from Zhao et al. found that weighted blankets produced a small but significant reduction in anxiety levels and a potential improvement in insomnia severity in sensitivity analysis). No serious adverse events were reported, though evidence quality was limited by high risk of bias (107). Exposure to neutral sensory environments was found to support emotional regulation and behavioral stability in individuals with heightened sensory sensitivities (108). Sensory integration interventions have been shown to reduce emotional and behavioral difficulties, including hyperactivity, aggression, anxiety, depression, somatization, attention and learning problems, atypical behaviors, and social withdrawal, in children with ASD (109). Evidence highlighted the potential efficacy of repeated Snoezelen room sessions in reducing the severity of ASD symptoms, particularly repetitive and stereotyped behaviors, as reflected in the Childhood Autism Rating Scale (CARS) scale (110). In the United Kingdom, it is estimated that approximately 40–90% of children diagnosed with ASD also meet criteria for at least one anxiety disorder, highlighting a substantial burden that also has significant repercussions on family life (111). Sensory integration interventions have been associated with improvements in a range of emotional and behavioral difficulties in children with ASD, including hyperactivity, aggression, anxiety, depression, somatization, attention and learning problems, atypical behaviors, and social withdrawal (109). In addition, evidence suggests that repeated sessions in a Snoezelen room may help reduce the severity of ASD symptoms, particularly repetitive and stereotyped behaviors, as reflected by improvements on the Childhood Autism Rating Scale (CARS) (110).

### 6.4 Theatre-based interventions

Theatre-based, peer-mediated intervention was associated with meaningful reductions in trait anxiety, likely driven by enhanced peer interaction and social engagement rather than changes in play behavior alone (112). Corbett et al. reported that a theatre-based intervention produced moderate-to-large improvements immediately post-treatment in social ability, communication symptoms, peer group play, facial memory both immediate and delayed, and theory of mind (113). At 2-month follow-up, benefits persisted for communication symptoms. Familiarity seemed to play an important role, as it reduced stress in ASD children. These findings provide preliminary evidence that theatre-based, peer-mediated interventions enhance social functioning, reduce social stress, and support cognitive–social processes in children with ASD (113).

### 6.5 Pharmacological therapy

Although no pharmacological treatments are currently approved for the core symptoms of Autism Spectrum Disorder (ASD), medications are frequently prescribed to address co-occurring psychiatric and behavioral symptoms, including anxiety, irritability, sleep disturbances, and ADHD-related symptoms (114, 115). Pharmacological treatment should generally be considered an individualized and adjunctive strategy that complements behavioral, psychosocial, educational, and family-based interventions, which remain central to the management of ASD and associated anxiety (116).

Pharmacological treatment typically targets specific symptom domains rather than ASD itself (117). When anxiety symptoms are clinically significant, Selective Serotonin Reuptake Inhibitors (SSRIs), including fluoxetine and sertraline, are among the most commonly prescribed medications, particularly when anxiety co-occurs with obsessive–compulsive symptoms (114, 115, 118). However, evidence regarding their efficacy and tolerability in children and adolescents with ASD remains mixed, and treatment decisions should be individualized. Other agents, such as buspirone and mirtazapine, have demonstrated potential anxiolytic effects in preliminary studies, while Serotonin–Norepinephrine Reuptake Inhibitors (SNRIs), including venlafaxine, may occasionally be considered in selected clinical circumstances (118, 119).

In contrast, medications such as risperidone and aripiprazole are primarily used to manage irritability, aggression, and severe behavioral dysregulation rather than anxiety itself (114, 115, 118). Similarly, co-occurring ADHD symptoms may be treated with stimulant medications, atomoxetine, or alpha-2 adrenergic agonists, depending on the individual’s clinical profile (114, 115, 118). A recent systematic review and network meta-analysis reported that several pharmacological agents, including atomoxetine, bumetanide, aripiprazole, and risperidone, may improve specific symptom domains in children and adolescents with ASD when compared with placebo; however, these findings relate to broader ASD-associated symptoms and comorbidities rather than anxiety specifically (120).

## 7 Non-modifiable vulnerability factors

For conceptual clarification, **Table 3** summarizes non-modifiable vulnerability markers and modifiable risk and protective factors that may represent potential intervention targets. The table categorizing these factors and interventions into: (1) non-modifiable vulnerability markers, (2) modifiable risk factors, (3) protective factors, and (4) therapeutic interventions.

**Table 3:** Classification of vulnerability markers, modifiable factors, protective factors, and intervention strategies associated with anxiety in children and adolescents with ASD.

Category	Examples
Non-modifiable vulnerability markers	Genetic susceptibility, family history, sex, menarche, age, puberty, IQ, VIQ
Modifiable risk factors	Sleep disturbances, physical inactivity, social isolation, bullying, emotion dysregulation, sensory overload
Protective factors	Family support, social support, school support, adaptive coping strategies, physical activity, resilience
Therapeutic interventions	CBT, sensory integration approaches, exercise interventions, psychotherapy, pharmacological interventions, digital interventions

CBT, cognitive behavioral therapy; IQ, intelligence Quotient; VIQ, verbal intelligence Quotient.

The non-modifiable vulnerability factors should not be considered intervention targets per se; however, they remain clinically important as they contribute to risk stratification and may inform individualized prevention, monitoring, and support strategies.

## 8 Discussion

Anxiety represents one of the most prevalent and clinically significant comorbidities in children and adolescents with Autism Spectrum Disorder (ASD), yet it remains under-recognized and insufficiently addressed in routine care (116). The present narrative overview, as the second part of a two-part series, extends the conceptual framework established in the depression-focused paper by examining modifiable risk and protective factors influencing anxiety, with an emphasis on clinically actionable and multidisciplinary strategies.

A central finding of this synthesis is that anxiety in ASD emerges from a complex interplay of biological, psychological, and environmental factors (2, 3). Sensory hypersensitivity, intolerance of uncertainty, social challenges, and environmental stressors appear to act as key drivers of anxious symptomatology. At the same time, these factors are not static; rather, they represent modifiable targets for intervention, reinforcing the importance of early identification and tailored management approaches.

Importantly, this overview highlights the growing—yet still fragmented—evidence base supporting protective and resilience-promoting factors. Interventions such as cognitive behavioral therapy (CBT), sensory integration strategies, structured physical activity, and psychosocial support demonstrate potential in reducing anxiety symptoms and improving emotional regulation (35, 44, 88–90, 108–110). However, the heterogeneity of study designs, small sample sizes, and variability in outcome measures limit the strength of conclusions and underscore the need for more standardized research. Among currently available interventions, CBT and structured physical activity appear to have the most consistent evidence supporting their use for anxiety-related symptoms in ASD, whereas digital interventions, sensory-based therapies, environmental modifications, and microbiota-targeted approaches should be considered promising but still evolving areas of investigation (9, 35, 44, 69–72, 82–84, 88–106).

Physical activity emerges as a particularly promising domain, with evidence suggesting beneficial effects on anxiety, emotional regulation, and overall functioning (91, 92, 94, 96). Similarly, family involvement and supportive environments play a crucial role in buffering stress and enhancing intervention outcomes (60). These findings support a shift toward holistic, ecosystem-based models of care, where the child is viewed within their broader social and environmental context.

Pharmacological approaches remain an adjunctive strategy, primarily targeting symptom clusters such as anxiety, irritability, or comorbid conditions. While medications such as SSRIs and other agents are commonly used in clinical practice, their efficacy and tolerability in ASD populations are variable, and their use should be carefully individualized and integrated within broader therapeutic frameworks (114, 115, 117, 118).

While several systematic reviews and meta-analyses have examined specific aspects of anxiety in ASD—such as prevalence, cognitive behavioral therapy, or technology-based interventions—existing literature remains largely fragmented and intervention-specific (13, 121–124). Few studies adopt a comprehensive, integrative perspective that simultaneously considers biological, psychosocial, environmental, and lifestyle-related factors. Moreover, protective and resilience-promoting factors remain underrepresented compared to risk-focused approaches.

To the best of our knowledge, no previous work has structured the synthesis of internalizing symptoms in ASD within a two-part, conceptually aligned framework addressing both depression and anxiety, while explicitly emphasizing modifiable protective factors and rehabilitation-oriented strategies.

A key contribution of this work lies in its balanced integration of risk and protective factors, moving beyond traditional deficit-focused models. However, the synthesis also reveals important gaps, including limited high-quality evidence on protective interventions, insufficient stratification across ASD subgroups, and a lack of longitudinal data to support causal inferences. Furthermore, the frequent overlap between anxiety and depression highlights the need for integrated models of internalizing psychopathology, rather than isolated condition-specific approaches.

Taken together, these findings reinforce the importance of early, personalized, and multidisciplinary interventions that simultaneously reduce risk exposure and strengthen resilience. This dual approach is essential for improving long-term mental health outcomes and quality of life in children and adolescents with ASD.

## 9 Limitations and strengths

Several limitations should be considered when interpreting the findings of this second part of the narrative overview focusing on anxiety in children and adolescents with Autism Spectrum Disorder (ASD). Firstly, the narrative design, while appropriate for integrating a heterogeneous and multidisciplinary body of evidence, does not follow the structured methodology of a systematic review. As such, the search strategy, study selection, and synthesis may be subject to selection bias, and no formal risk-of-bias assessment or quantitative meta-analysis was conducted.

Secondly, the included literature is characterized by substantial heterogeneity in terms of study design, sample characteristics, outcome measures, and intervention protocols. This variability limits direct comparability across studies and constrains the ability to draw definitive conclusions regarding the magnitude and consistency of effects, particularly for specific interventions targeting anxiety in ASD.

Thirdly, although this work aimed to emphasize both risk and protective factors, the available evidence on protective influences—especially those related to lifestyle, rehabilitation, and psychosocial interventions—remains fragmented and, in some cases, preliminary. Consequently, certain findings should be interpreted with caution, particularly where evidence is derived from small samples, non-randomized designs, or studies not specifically focused on ASD populations.

Fourthly, ASD was necessarily treated at a broad level, and the synthesis does not fully account for intra-spectrum variability, including differences in age, cognitive functioning, language abilities, sex, and comorbid conditions. This may limit the generalizability of the findings and highlights the need for more stratified and longitudinal research.

Finally, while anxiety is the primary focus of this second part, its frequent co-occurrence with depression and other internalizing symptoms introduces conceptual and clinical overlap that may not be entirely disentangled within the current framework. Despite structuring this work as part of a two-part series, some degree of overlap between constructs is inevitable.

Despite these limitations, the narrative and integrative nature of this overview allows for a broad, clinically relevant synthesis of current evidence, highlighting key gaps and supporting the development of multidisciplinary, prevention-oriented approaches for managing anxiety in children and adolescents with ASD.

This narrative overview presents several notable strengths. First, it adopts a comprehensive and integrative perspective, synthesizing evidence across biological, psychological, environmental, and rehabilitative domains to reflect the multifactorial nature of anxiety in children and adolescents with Autism Spectrum Disorder (ASD). Second, by explicitly examining both modifiable risk and protective factors, this work moves beyond traditional deficit-oriented models and contributes to a more balanced, resilience-focused framework. Third, the study incorporates findings from high-quality sources, including systematic reviews, meta-analyses, and key clinical studies, while maintaining a clinically meaningful interpretation of heterogeneous data. Fourth, its two-part structure, developed in continuity with the companion paper on depression, allows for both targeted analysis and conceptual coherence, acknowledging the frequent co-occurrence and shared mechanisms of internalizing symptoms in ASD. Finally, the emphasis on rehabilitation, physical activity, psychosocial interventions, and lifestyle-based strategies enhances the translational value of the review, offering practical insights for multidisciplinary care and supporting prevention-oriented approaches in this vulnerable population.

## 10 Key points

Several key considerations emerge regarding the modulation of risk and protective factors for anxiety in children and adolescents with ASD:

- Anxiety is Highly Prevalent and Often Overlapping with Other Conditions: Anxiety disorders affect a substantial proportion of youth with ASD and frequently co-occur with depression and other internalizing symptoms, complicating both identification and management.
- Sensory and Environmental Triggers Play a Central Role: Heightened sensory sensitivity, intolerance of uncertainty, and environmental stressors (e.g., unpredictability, social demands) are major contributors to anxiety in ASD, highlighting the importance of structured, predictable, and supportive environments.
- Protective Factors Are Underexplored but Clinically Actionable: Interventions such as cognitive-behavioral therapy (CBT), sensory integration strategies, and supportive psychosocial environments show promise in reducing anxiety, although evidence remains heterogeneous.
- Physical Activity and Structured Interventions Support Emotional Regulation: Exercise-based approaches—including aerobic activity, group exercise, and mind–body practices—demonstrate beneficial effects on anxiety reduction and overall emotional wellbeing.
- Family and Social Context Strongly Influence Anxiety Outcomes: Parental involvement, psychoeducation, and stable support systems play a crucial role in buffering stress, improving coping strategies, and enhancing treatment adherence.
- Personalized, Multidisciplinary Care is Essential: Given the heterogeneity of ASD, interventions must be tailored to individual profiles, integrating behavioral, rehabilitative, environmental, and, when appropriate, pharmacological strategies.
- Early Identification and Prevention Are Critical: Timely recognition of anxiety symptoms and early implementation of targeted interventions can significantly reduce long-term psychological burden and improve quality of life.

## 11 Conclusions

This second part of the narrative overview provides a comprehensive synthesis of modifiable factors associated with anxiety in children and adolescents with Autism Spectrum Disorder, complementing the depression-focused findings presented in Part I. The evidence supports a shift toward integrative, resilience-oriented models of care, in which anxiety is understood not only as a consequence of vulnerability but also as a modifiable outcome influenced by environmental, behavioral, and therapeutic factors.

While current research highlights the potential of interventions such as cognitive behavioral therapy, physical activity, sensory-based approaches, and family-centered care, the overall evidence base remains heterogeneous and, in some areas, preliminary. Future research should prioritize methodological consistency, longitudinal designs, and stratified analyses to better capture the complexity and variability of ASD populations.

Ultimately, improving anxiety outcomes in ASD requires a multidisciplinary and individualized approach, integrating clinical, rehabilitative, and psychosocial strategies. By advancing a more balanced understanding of both risk and protective pathways, this work contributes to the development of practical, prevention-oriented frameworks that can inform clinical practice and support the mental well-being of this vulnerable population.

## Supplementary material

Supplementary Table 1 Summary of the literature search strategy.

Supplementary Figure 1 Searches conducted for the narrative overview. *Literature identification and selection process used for the narrative overview. Searches were conducted iteratively across PubMed, PubMed Central (PMC), Scopus, and Google Scholar between April 2024 and October 2025 using combinations of ASD-, anxiety-, mental health-, risk factor-, protective factor-, and intervention-related keywords and MeSH terms. Retrieved studies underwent title, abstract, and full-text assessment by multiple reviewers and were selected according to predefined eligibility criteria, methodological robustness, relevance to the review objectives, and contribution to the conceptual framework. The final narrative synthesis incorporated evidence from 110 studies.
