# Supplementary material for: From risk to resilience: a narrative overview of modifiable factors influencing anxiety in children with autism spectrum disorder (part II)
Source: Front Psychiatry. 2026 Jul 17;17:1872455. doi: 10.3389/fpsyt.2026.1872455 (PMC13424484; doi:10.3389/fpsyt.2026.1872455)
Supplement: Supplementary Table 1 — Summary of the literature search strategy. [file Supplementaryfile1.pdf]

Supplementary Material

Supplementary Table S1. Summary of the Literature Search Strategy

Search Date	Database(s)	Search Terms / Strategy	Filters Applied	Search retrieval counts
17 Apr 2024	PubMed, PMC	"physical activity level" AND "autism" OR "autism"	clinical trial, clinical study, observational study, randomized controlled trial	367
17 Apr 2024	PubMed, PMC	"physical activity level" AND "autism spectrum disorder"	clinical trial, clinical study, observational study, randomized controlled trial	274
17 Apr 2024	PubMed, PMC	"decreased physical activity level" AND "autism spectrum disorder"	clinical trial, clinical study, observational study, randomized controlled trial	46
17 Apr 2024	PubMed, PMC	"youth" AND "stress" AND "anxiety" AND "autism spectrum disorder"	clinical trial, clinical study, observational study, randomized controlled trial	15
25 Oct 2024	PubMed, PMC	"youth" AND "stress" AND "anxiety" AND "autism spectrum disorder"	Additional article types including systematic reviews and meta-analyses	77
25 Oct 2024	PubMed, PMC, Scopus, Google Scholar	"autism spectrum disorder" "OR "ASD"AND "anxiety" AND "depression"	clinical trial, clinical study, observational study, randomized controlled trial; systematic reviews and meta-analyses; abstract available	3,487
21 Jun 2025	PubMed, PMC, Scopus, Google Scholar	Updated search using "ASD-", "autism spectrum disorder", "anxiety-", "resilience-", "risk factor-", "protective factor-," and "intervention-related" keywords and MeSH terms	Eligibility criteria applied according to narrative review protocol	242
21 Jul 2025	PubMed, PMC, Scopus, Google Scholar	Updated search using "ASD-", "autism spectrum disorder", "anxiety-", "resilience-", "risk factor-", "protective factor-", and "intervention-related" keywords and MeSH terms	Eligibility criteria applied according to narrative review protocol	247

Search Date	Database(s)	Search Terms / Strategy	Filters Applied	Search retrieval counts
18 Sep 2025	PubMed, PMC, Scopus, Google Scholar	Updated search using "ASD-", "autism spectrum disorder", "anxiety-", "resilience-", "risk factor-", "protective factor-", and "intervention-related" keywords and MeSH terms	Eligibility criteria applied according to narrative review protocol	250
05 Oct 2025	PubMed, PMC, Scopus, Google Scholar	Final update using keywords and MeSH terms: autism spectrum disorder (ASD), anxiety, anxiety disorders, internalizing symptoms, risk factors, protective factors, modifiable factors, mental health, mental health disorders (MHD), resilience, prevention, psychosocial interventions, rehabilitation, rehabilitative strategies, sensory regulation, lifestyle interventions, anxiety in children and adolescents with ASD, and children and adolescents	Inclusion and exclusion criteria applied; duplicate and irrelevant records removed through title, abstract, and full-text screening	Studies contributing to final narrative synthesis (n = 110)

* Searches were conducted iteratively between April 2024 and October 2025 and were refined throughout the review process. As several search strategies overlapped in scope and retrieved some of the same publications, the reported retrieval counts should not be summed to estimate the total number of unique records identified. Studies were selected based on the predefined inclusion and exclusion criteria, methodological robustness, relevance to the conceptual framework, and contribution to understanding risk and protective factors associated with anxiety in children and adolescents with ASD. The final narrative synthesis included 110 studies. Additional references were cited to provide methodological, contextual, and background information.

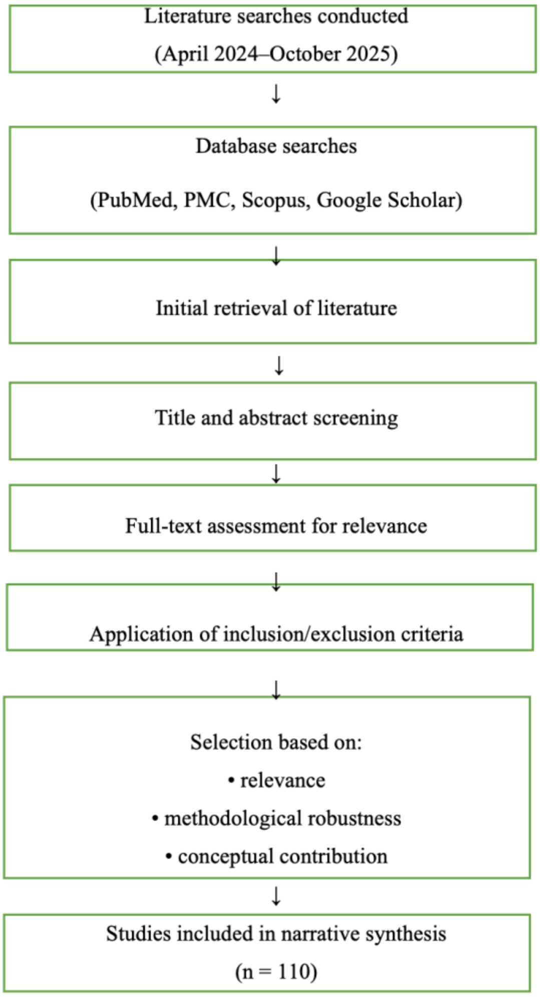

Supplementary Figure S1. Searches Conducted for the Narrative Overview

*Literature identification and selection process used for the narrative overview. Searches were conducted iteratively across PubMed, PubMed Central (PMC), Scopus, and Google Scholar between April 2024 and October 2025 using combinations of ASD-, anxiety-, mental health-, risk factor-, protective factor-, and intervention-related keywords and MeSH terms. Retrieved studies underwent title, abstract, and full-text assessment by multiple reviewers and were selected according to predefined eligibility criteria, methodological robustness, relevance to the review objectives, and contribution to the conceptual framework. The final narrative synthesis incorporated evidence from 110 studies.
